# Study on Residual Strength of Pipelines with Single-Point Uniform Corrosion Defects Under Internal Pressure Loading

**DOI:** 10.3390/ma19112389

**Published:** 2026-06-03

**Authors:** Lihua Chen, Guoxing Yu, Die Liu, Youjia Zhang, Shuqin Zheng, Xu Wang, Yanru Wang, Lei Zhou

**Affiliations:** 1 School of Civil Engineering, Chongqing Vocational Institute of Engineering, Chongqing 402260, China; clh@cqvie.edu.cn; 2 School of Civil Engineering, Chongqing Jiaotong University, Chongqing 400074, China; liudie111@mails.cqjtu.edu.cn (D.L.); xuwang@cqjtu.edu.cn (X.W.); lixuezl@tju.edu.cn (L.Z.); 3 Chief Engineer Office, Chongqing CISDI Engineering Consulting Co., Ltd., Chongqing 400013, China; guoxing.yu@cisdi.com.cn; 4 School of Civil Engineering and Architecture, Northeast Electric Power University, Jilin 132012, China; 5 Key Laboratory of Intelligent Lifeline Protection and Emergency Technology for Resident ATY, Wenzhou University of Technology, Wenzhou 325000, China; 6 School of Civil Engineering, Taizhou University, Jiaojiang 318000, China; yanrupiaoyang@163.com

**Keywords:** oil and gas pipeline, uniform corrosion, single-point defect, finite element analysis, failure pressure, residual strength

## Abstract

Steel pipelines for oil and gas transportation serve as the lifeline of energy conveyance, and their long-term safe operation constitutes a crucial safeguard for energy security. Nevertheless, in complex service environments, local defects formed on the inner pipe wall due to medium corrosion have emerged as a prominent hidden danger endangering pipeline integrity. Accurate evaluation of the residual strength of pipelines with corrosion defects is not only the technical foundation for ensuring the safe operation of pipelines, but also the key basis for formulating scientific maintenance strategies and prolonging the service life of pipelines. Taking three grades of steel pipelines (X52, X65 and X80), which represent the typical strength grades commonly used in long-distance oil and gas transmission pipelines, as the research objects, this paper establishes a three-dimensional finite element model of single-point uniform corrosion defects considering the nonlinear material behavior, and systematically investigates the influence laws of geometric parameters (depth, length and width) of corrosion defects on the failure pressure of pipelines under the action of monotonic internal pressure load. The accuracy of the proposed finite element model is verified by comparison with the test data from thirteen groups of full-scale burst experiments. On the basis of parametric analysis results, an explicit and high-precision predictive model for failure pressure is developed. The research findings reveal that corrosion depth acts as the dominant factor affecting pipeline failure pressure with a distinctly nonlinear influence characteristic: the load-bearing capacity of pipelines drops drastically when the relative depth d/t exceeds 0.6, where d is the corrosion depth and t is the pipe wall thickness. There exists a critical value for the impact of corrosion length, beyond which its weakening effect on failure pressure tends to level off. Within the commonly encountered engineering range (20~100°), corrosion width exerts a negligible influence on pipeline failure pressure and thus can be overlooked in engineering evaluation. In comparison with conventional industry assessment methods such as ASME B31G, DNV RP-F101, PCORRC and SY/T 6151, the newly established predictive model features higher prediction accuracy and broader applicability, which provides on-site engineers with a powerful theoretical tool and practical formula for the rapid and accurate evaluation of the residual strength of corroded pipelines.

## 1. Introduction

Pipeline transportation plays an irreplaceable role in the global energy strategy by virtue of its high efficiency, economic benefits and operational safety. According to statistics, the total mileage of long-distance oil and gas pipelines worldwide is estimated to have exceeded 2 million kilometers by the end of 2024, a large proportion of which have been in continuous operation for decades and thus gradually entered the aging stage with a marked rise in accident risks [1,2]. Statistical reports on pipeline failures clearly indicate that corrosion is the primary or secondary cause of severe accidents such as pipeline leakage and explosion, accounting for as high as 20% to 24% of all incidents. As the “energy arteries” of cities, buried urban oil and gas pipelines have their operational safety directly linked to public security and social stability [3,4,5]. Therefore, conducting accurate residual strength assessment for pipelines with corrosion defects, predicting their failure pressure [6,7,8,9], and formulating rational inspection, maintenance and replacement plans on this basis are of great social and economic significance.

Traditional assessment of pipeline residual strength mainly relies on empirical formulas or simplified analytical models generalized from a large volume of test data, such as the ASME B31G criterion and DNV RP-F101 recommended practice. Despite their extensive application in engineering practice, these methods tend to yield conservative evaluation results due to their foundation on elastic mechanics or simplified plastic assumptions, as well as the common idealized treatment of defect geometries. Such conservatism may, on the one hand, lead to unnecessary pipeline repair or replacement and cause a waste of resources; on the other hand, it might also cover up the actual risks posed by certain complex defects. With the rapid development of computer technology and numerical calculation methods, the finite element method has evolved into a powerful tool for investigating the mechanical behaviors of complex engineering structures. It is capable of accurately accounting for the nonlinear constitutive relations of materials, complex geometric shapes and boundary conditions, thereby deriving the stress–strain field and ultimate bearing capacity that are more consistent with the actual situation.

At present, numerous scholars have conducted extensive research on the residual strength of corroded pipelines using the finite element method and achieved fruitful outcomes. Early studies primarily focused on verifying the feasibility of the FEM approach and its consistency with experimental results through three-dimensional solid modeling. With the deepening of research, scholars began to systematically analyze the influence laws of individual defect parameters (e.g., depth, length) on failure pressure. The interaction between multiple corrosion defects was further investigated, and criteria for treating closely spaced defects as a single equivalent flaw were proposed [10]. More recently, the stress concentration interaction between adjacent defects was analyzed [11], and the crack-like behavior of deep corrosion flaws was captured using XFEM [12], both providing refined insight into failure mechanisms. Nevertheless, most studies have been targeted at pipelines of specific steel grades or dimensions, lacking a systematic comparative analysis under a unified framework. In addition, existing research has paid insufficient attention to the effect of defect width, and the conclusion that “the influence of width is negligible” has usually been drawn based on individual cases, without systematic parametric verification. In terms of failure pressure prediction models, a comprehensive evaluation of multiple burst strength models was conducted, and the critical role of the failure criterion was highlighted [13]. Despite these advances, the forms of existing models are often relatively complex or their application scopes are limited, which hinders their popularization and widespread use in engineering practice.

In summary, this paper aims to make up for the deficiencies of existing studies through systematic finite element parametric analysis. The specific objectives are as follows: (1) Establish refined finite element models applicable to three pipeline grades with different strength levels, namely X52, X65 and X80, and conduct strict verification by using full-scale burst test data; (2) systematically quantify the independent and coupled effects of three key geometric parameters (corrosion depth, length and width) on pipeline failure pressure, and clarify their influence mechanisms and variation laws; (3) based on a large number of numerical calculation results, develop an explicit failure pressure prediction model with a simple form, reliable accuracy and clear physical significance, so as to provide a directly applicable assessment tool for engineering practice.

## 2. Finite Element Modeling Methodology and Validation

### 2.1. Geometric Model Establishment and Basic Assumptions

**Figure 1:**
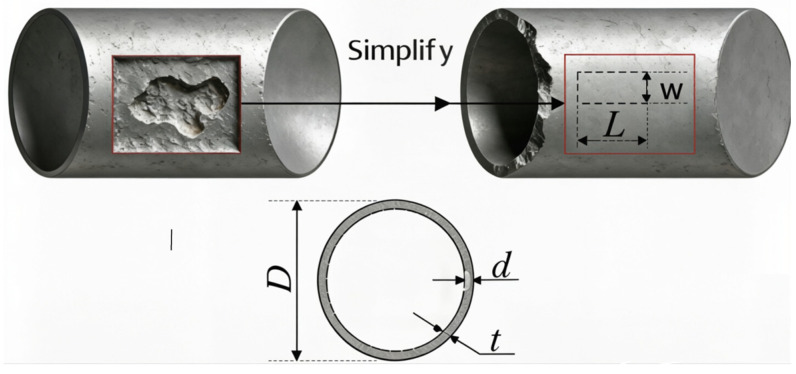
Schematic diagram of the defect.

To balance computational accuracy and efficiency, reasonable simplifications are applied to the practical engineering problem in this study. A pipeline segment with corrosion defects is selected for modeling, and the axial length of the model is set to more than 1.5 times the outer diameter D of the pipeline [14], where D is the pipe outer diameter and t is the pipe wall thickness, to eliminate boundary effects. Actual corrosion pits are mostly irregular in shape; for the convenience of parametric research and to enhance model universality, the irregular corrosion area is simplified into a rectangular defect using the equal area method [15,16] (Figure 1). The rectangular defect is defined by three parameters: the length L along the pipeline axial direction, the width w along the pipeline circumferential direction (expressed by the central angle), and the depth d along the wall thickness direction.

To focus on the effects of internal pressure loads and corrosion defects themselves, the following reasonable assumptions are made in this study:

(1) The interaction between buried pipelines and the surrounding soil, including soil constraints and external earth pressure, is neglected.
(2) Thermal stress induced by temperature changes and secondary loads such as pipeline self-weight and seismic loads are not considered.
(3) Pipeline materials are assumed to be homogeneous and isotropic with no initial residual stress.
(4) The pressure exerted by the conveying medium inside the pipeline on the inner wall is regarded as uniform static pressure, and the hydrodynamic effects are ignored.

### 2.2. Material Constitutive Model and Parameters

Under internal pressure loading, the regions adjacent to corrosion defects in pipeline steel typically enter the plastic stage. Therefore, adopting a constitutive model that can accurately characterize the plastic behavior of materials is the key to obtaining reliable results. In this study, the true stress–true strain curves converted from the engineering stress–strain curves of uniaxial tensile tests are employed as the material constitutive model, following the standard conversion relations [17] (Equations (1) and (2)). This model can accurately reflect the hardening behavior of materials in the plastic stage; it not only avoids the trouble of fitting the hardening exponent required by the Ramberg–Osgood model, but also is more consistent with actual conditions than the ideal elastic–plastic model.

(1) $$\varepsilon_{true}=In(1+\varepsilon_{nom})$$

(2) $$\sigma_{true}=\sigma_{nom}(1+\varepsilon_{nom})$$

where

$\varepsilon_{true}$—true strain;
$\sigma_{true}$—true stress (MPa);
$\varepsilon_{nom}$—nominal strain;
  $\sigma_{nom}$—nominal stress (MPa).

**Figure 2:**
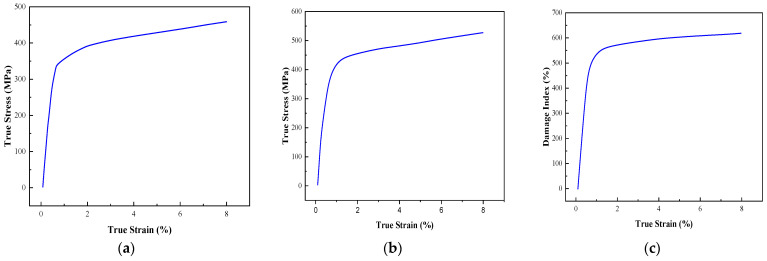
The true stress–strain curves. (**a**) X52; (**b**) X65; (**c**) X8.

In this study, three typical grade steels of X52, X65 and X80 pipelines specified in the API 5L standard are selected as the research objects, with their geometric dimensions and mechanical property parameters presented in Table 1. Detailed stress–strain data of these three steels were obtained from published experimental studies and material databases: the X52 data were taken from the tensile test results reported by Law and Bowie [18], the X65 data from the characterization work by Tanguy et al. [19], and the X80 data from the mechanical property study by Shin et al. [20]. On the basis of these data, the true stress–strain curves were plotted [21] (Figure 2). The elastic modulus of all materials is taken as 206 GPa with a Poisson’s ratio of 0.3.

### 2.3. Meshing Strategy and Boundary Conditions

The ABAQUS/Standard module is adopted for the numerical analysis. The pipeline solid model is meshed with eight-node linear hexahedral reduced integration elements (C3D8R), which can effectively avoid shear locking and ensure high computational efficiency in simulating elasto-plastic problems with large deformations.

**Figure 3:**
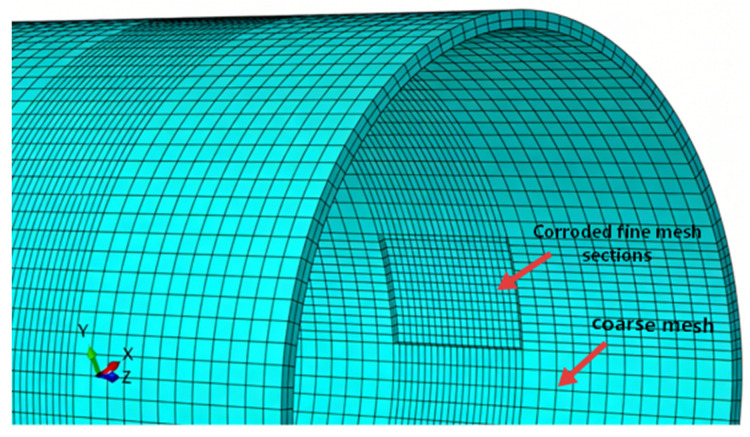
Mesh distribution of uniform corrosion.

The quality of mesh generation directly affects the accuracy and convergence of numerical calculation results. For defect-containing structures, severe stress concentration and gradient variation exist in the defect zone, which requires a denser mesh to capture its mechanical response. A zoned meshing technique is adopted in this study: a refined mesh (with a size of approximately 15 mm × 15 mm) is applied in the corrosion defect zone and its surrounding areas, while a relatively coarse mesh (with a size of approximately 25 mm × 25 mm) is used in the pipeline regions far from the defect (Figure 3). Mesh sensitivity analysis is performed to verify that this meshing scheme ensures calculation accuracy while maintaining an acceptable computational cost.

**Figure 4:**
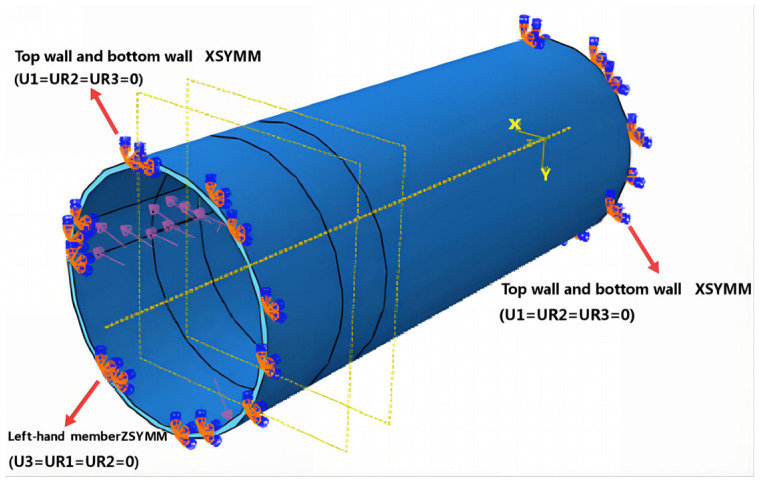
Schematic of boundary condition setup.

The boundary conditions are defined to simulate a segment of a pipeline in long-distance laying. Axial (X-direction) displacement constraints (U1 = 0) are imposed on all nodes at one end of the pipeline to balance the axial force induced by internal pressure, while free contraction and deformation of the pipeline at this end face are allowed (UR2 and UR3 are unconstrained). The other end and the entire outer surface of the pipeline are set to be fully free (Figure 4). In terms of loading, a uniformly distributed hydrostatic pressure is applied on all element faces of the pipeline inner wall, and the pressure value is monotonically increased from zero until the pipeline undergoes failure.

### 2.4. Failure Criterion

To determine the failure pressure of a pipeline, an explicit failure criterion is required. This study adopts the plastic failure criterion, which is widely recognized in the research on corroded pipelines. According to this criterion, a pipeline experiences failure (rupture) when the maximum von Mises equivalent stress on the minimum cross-section of the corrosion defect zone reaches the ultimate tensile strength of the pipeline material, and the applied internal pressure at this moment is defined as the failure pressure. This criterion takes into account the strain hardening stage of the material after yielding, and is more consistent with the actual physical process of pipeline burst failure than the elastic limit criterion or plastic limit criterion that are solely based on yield strength [22,23,24]. This study adopts the plastic failure criterion [25], under which the von Mises stress is calculated as Equation (3). In the post-processing of ABAQUS, the failure pressure is determined by plotting the stress distribution along a specific path or monitoring the stress history of the elements at the defect bottom, which corresponds to the moment when the maximum equivalent stress reaches the ultimate tensile strength.

(3) $$\sigma_{s}=\sqrt{\frac{1}{2}{\left[{\left(\sigma_{h}-\sigma_{r}\right)}^{2}+{\left(\sigma_{r}-\sigma_{l}\right)}^{2}+(\sigma_{l}-\sigma_{h})\right]}^{2}}$$

where

$\sigma_{h}$—Circumferential stress (MPa);
$\sigma_{r}$—Radial stress (MPa);
$\sigma_{l}$—Axial stress (MPa).

### 2.5. Model Validation

Typically, full-scale burst testing of pipelines is the most reliable means of validating the results of the proposed finite element model. In such a test, a pipe segment containing a pre-machined corrosion defect is sealed at both ends, filled with a liquid pressurizing medium, and subjected to a monotonically increasing internal pressure until the pipe wall ruptures. The failure pressure and the strain evolution at the defect are recorded simultaneously to obtain the ultimate load-bearing capacity of the pipeline. However, due to the high cost and significant risks of burst tests, such testing was not conducted in this study. Therefore, the reliability of the finite element simulation model was verified by referring to real burst tests performed by other researchers under the same internal pressure conditions as those considered herein. A total of 13 sets of full-scale burst test data for corroded pipelines were collected [26,27,28], as presented in Table 2, covering three pipeline steel grades: X60, X65, and X80.

The established finite element model was used to perform simulation calculations for these 13 sets of cases, and the predicted failure pressure values obtained therefrom were compared with the experimentally measured values. Meanwhile, to demonstrate the superiority of the method proposed in this study, four commonly used industry standards and methods, namely the modified ASME B31G [29], DNV RP-F101 [30], PCORRC [31] and SY/T 6151 [32], were also adopted to conduct comparative calculations for the same cases.

**Figure 5:**
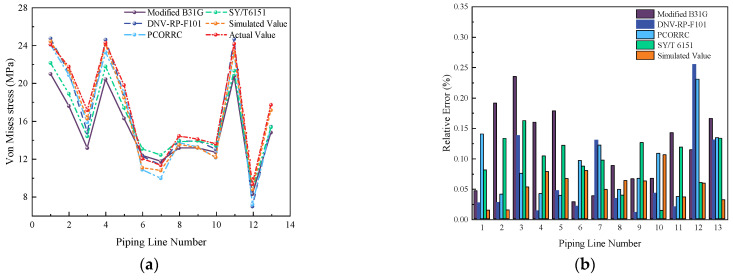
Comparison curves of failure pressure. (**a**) Comparison curves; (**b**) error analysis.

The validation results are presented in Table 3 and Figure 5. For the finite element method (FEM), the mean relative error between the predicted results and the experimental values is 5.49%, with a maximum relative error of 7.8%, and all predicted values fall within the ±10% error band of the experimental data. In contrast, the mean relative errors of the four traditional methods range from 7.84% to 11.95%, with some predicted values showing significant dispersion and an obvious conservative tendency. These findings indicate that the settings of the finite element model established in this study, including those for the material constitutive relation, meshing strategy, boundary conditions and failure criterion, are rational and accurate, and the model is capable of being applied to subsequent systematic parametric studies.

## 3. Analysis of the Influence Law of Single-Point Corrosion Defect Parameters

### 3.1. Influence Mechanism and Quantitative Analysis of Corrosion Depth

**Figure 6:**
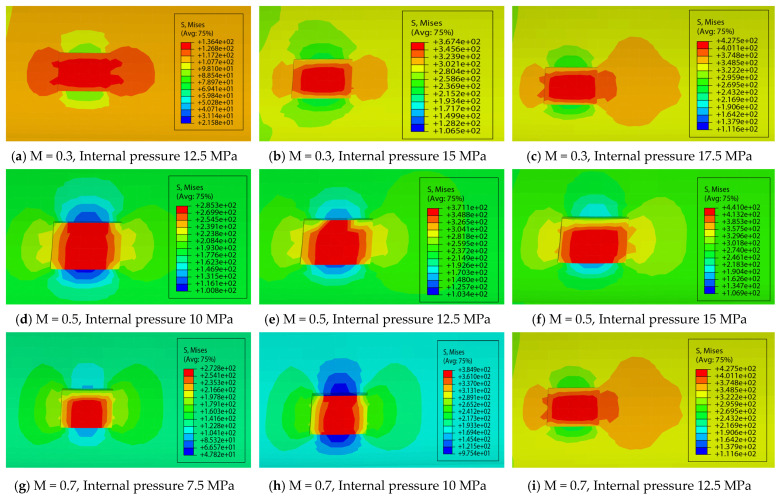
Von Mises stress nephograms of X52 grade pipeline.

Corrosion depth is the most direct parameter for measuring the material loss of pipeline walls. To investigate its effects systematically, for each steel grade of pipeline, the corrosion length and width were fixed while the relative corrosion depth d/t was varied from 0.2 to 0.8 with an interval of 0.1, generating a total of 21 calculation cases. Figure 6 presents the von Mises stress nephograms of the X52 pipeline under different corrosion depths and internal pressures. It can be clearly observed that the stress concentration zone is consistently located at the bottom of the corrosion defect. As the internal pressure increases, the high-stress zone expands from the defect bottom to the surrounding areas. At a shallow corrosion depth (d/t = 0.3), the range of the high-stress zone is relatively small; in contrast, at a large corrosion depth (d/t = 0.7), the stress at the defect bottom approaches the yield strength of the material even under a relatively low internal pressure, with a much wider distribution of the high-stress zone.

**Figure 7:**
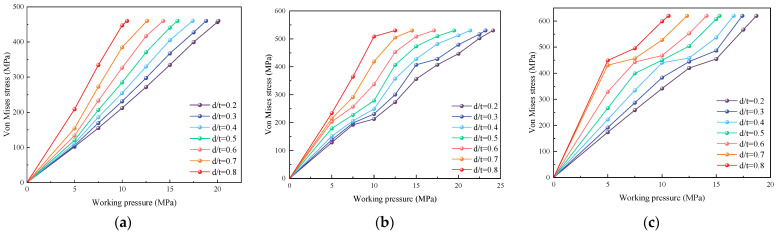
Axial fitting results. (**a**) X52; (**b**) X65; (**c**) X80.

Figure 7 plots the variation curves of the maximum von Mises stress in the defect zone with corrosion depth for the three steel grade pipelines under different internal pressures. All curves exhibit a monotonically increasing trend of stress with the rise in corrosion depth. Taking the X52 pipeline under an internal pressure of 10 MPa as an example, the maximum stress rises from 231.1 MPa to 254.8 MPa with an increase of 10.3% when d/t increases from 0.3 to 0.4; in contrast, the maximum stress surges from 371.2 MPa to 458.5 MPa with a substantial increase of 23.5% when d/t rises from 0.6 to 0.7. This indicates that the influence of corrosion depth is nonlinear, and the stress growth induced by a unit increase in depth becomes more drastic at larger corrosion depths. The X65 and X80 pipelines exhibit an entirely similar trend.

**Figure 8:**
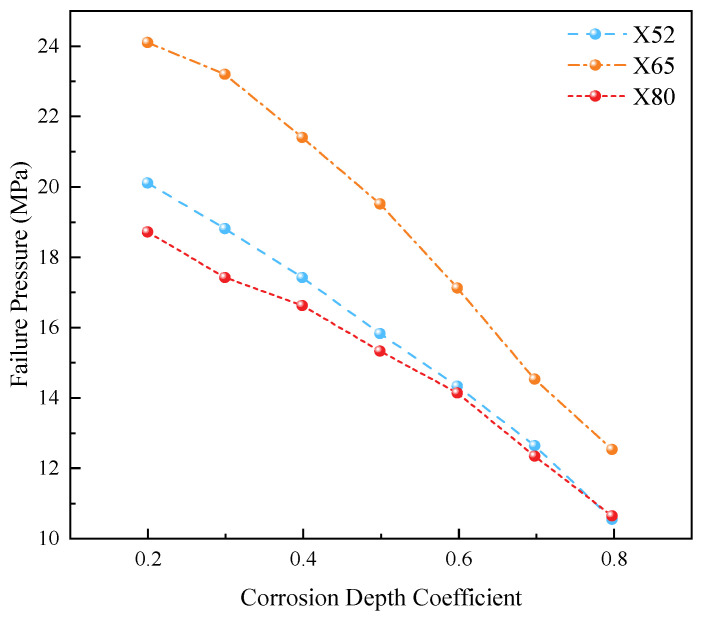
Relationship between defect depth and failure pressure.

The pressure at failure (i.e., the failure pressure) under each working condition was extracted and plotted in Figure 8. This figure intuitively reveals the law that the failure pressure decreases with the increase in corrosion depth. Notably, the descending curve is not a straight line but exhibits a characteristic of a slow decrease followed by a rapid drop. When d/t < 0.6, the curve declines relatively gently; in contrast, the slope of the curve increases significantly when d/t > 0.6, indicating an accelerated attenuation of the residual strength of the pipeline. For instance, for the X52 pipeline, the failure pressure decreases by approximately 1.3 MPa as d/t increases from 0.2 to 0.3, whereas it drops by a substantial 2.1 MPa when d/t rises from 0.7 to 0.8. In engineering practice, d/t = 0.8 is commonly regarded as the critical value requiring immediate repair, and this study verifies the rationality of this regulation from a mechanical perspective: beyond this depth, the load-bearing capacity of the pipeline becomes extremely fragile, and a slight increase in depth may lead to catastrophic failure.

### 3.2. Influence Mechanism and Critical Phenomenon of Defect Length

**Figure 9:**
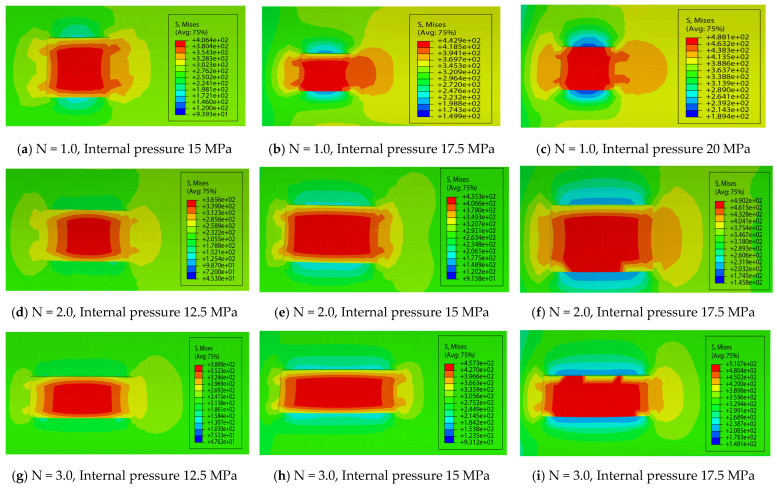
Von Mises stress nephograms of X65 grade pipeline.

Defect length determines the axial extension range of defects. The relative defect depth d/t was fixed at 0.4 and the corrosion width w at 20°, with the relative defect length $\left(N=L/\sqrt{Dt}\right)$ varied from 0.5 to 5.0 to investigate the influence of defect length. The stress nephograms in Figure 9 show that for short defects (e.g., N=1.0), stress concentration occurs mainly at the two ends (axial boundaries) of the defect; for long defects (e.g., N=3.0), the stress at the middle of the defect also remains at a high level, and an almost uniform high-stress zone is formed along the entire bottom of the defect.

**Figure 10:**
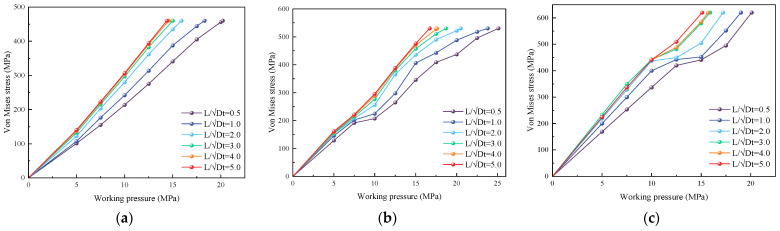
Effect of corrosion defect length coefficient on von Mises stress of pipelines. (**a**) X52; (**b**) X65; (**c**) X80.

**Figure 11:**
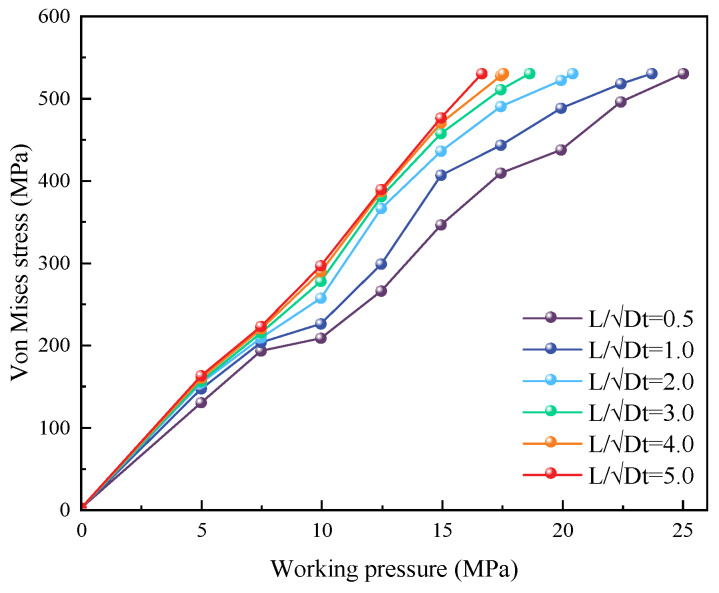
Relationship between defect length and failure pressure.

The stress–length relationship curves in Figure 10 indicate that under the same internal pressure, the maximum stress in the defect zone increases with the rise in defect length, yet the growth rate slows down gradually. The failure pressure–length relationship curves in Figure 11 reveal an important phenomenon: the failure pressure decreases as the defect length increases, but the declining trend becomes extremely gentle and almost reaches a stable value when the length exceeds a certain critical value, which is approximately around $L/\sqrt{Dt}=3.0$. Taking the X65 pipeline as an example, the failure pressure drops by about 2.5 MPa when it increases from 0.5 to 1.0; in contrast, it only decreases by approximately 0.3 MPa when it rises from 3.0 to 4.0.

The mechanical mechanism underlying this phenomenon can be explained as follows: for short defects, the boundary effect at both ends of the defect is prominent, and the material in the middle zone of the defect is strongly supported by the surrounding intact material, resulting in a relatively low average stress level. As the defect extends, such supporting effect weakens, and the stress in the defect midsection rises rapidly to a level close to that at both ends, leading to a reduction in the overall load-bearing capacity. When the defect is sufficiently long, an independent weakened zone is formed in the defective area, whose load-bearing capacity is mainly determined by the minimum residual wall thickness at this location. Further increasing the defect length merely extends this weakened zone, while the load-bearing capacity per unit length remains unchanged, thus the total failure pressure tends to stabilize. This finding provides important guidance for the assessment of long corrosion defects: for extremely long defects, it is unnecessary to accurately measure their full length, and only the zone with the maximum corrosion depth needs to be focused on.

### 3.3. Evaluation of the Influence of Defect Width

**Figure 12:**
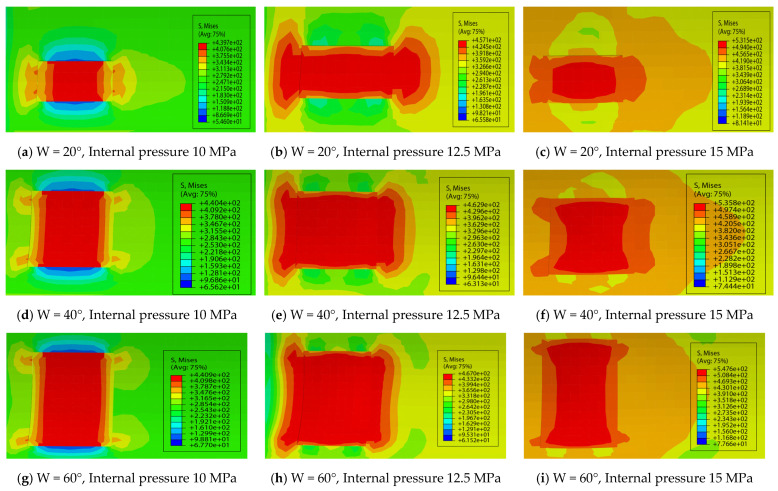
Von Mises stress nephograms of X82 grade pipeline.

Defect width defines the circumferential expansion angle of defects. With the defect depth and length fixed, the defect width w was varied from 20° to 100°. The stress nephograms in Figure 12 show that stress concentration is mainly distributed along the circumferential centerline of the defect, and the variation in defect width does not alter this fundamental distribution pattern.

**Figure 13:**
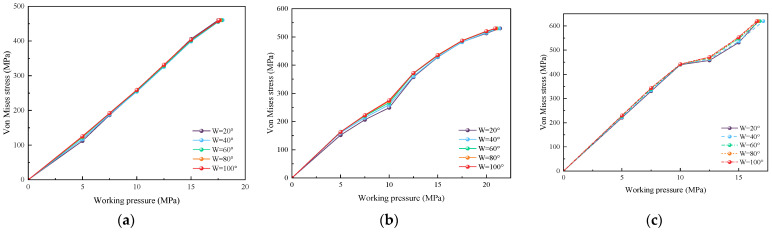
Effect of corrosion defect width coefficient on von Mises stress of pipelines. (**a**) X52; (**b**) X65; (**c**) X80.

**Figure 14:**
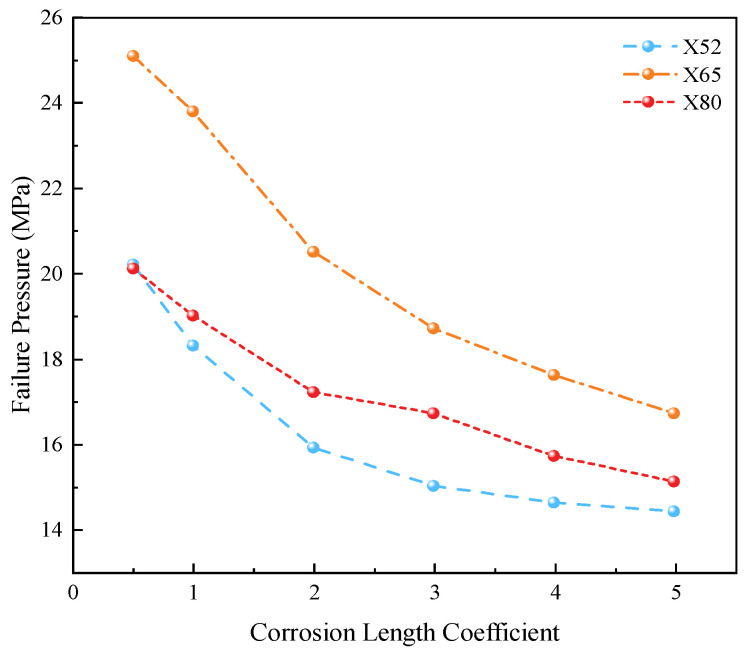
Relationship between defect length and failure pressure.

The quantitative analysis results in Figure 13 and Figure 14 clearly demonstrate that corrosion width exerts a negligible influence on the maximum stress in the defect zone and the failure pressure of the pipeline. The fluctuation range of failure pressure for the three steel grade pipelines under different defect widths is less than 0.5%. For instance, the failure pressure of the X80 pipeline at a corrosion width of 20° and 100° differs by only 0.2 MPa, with a relative error of less than 1% compared to its nominal failure pressure.

This conclusion is consistent with the primary stress-bearing characteristics of pipelines under internal pressure. For thin-walled cylinders under internal pressure, the circumferential stress is twice the axial stress and acts as the dominant driving force for failure. The depth and length of corrosion defects directly affect the effective cross-sectional area bearing the circumferential stress (i.e., the residual wall thickness and load-bearing length), whereas the defect width (in non-penetrating cases) mainly influences the local flexural stiffness and exerts a minimal effect on the circumferential membrane stress. It is therefore reasonable and conservative to neglect the influence of corrosion width as a simplification in engineering assessment. This greatly streamlines the processing of field inspection data, as accurately measuring the irregular corrosion width is often extremely challenging.

## 4. Establishment and Application of the Failure Pressure Prediction Model

### 4.1. Deduction of the Model Form

Based on the aforementioned parameter analysis, the failure pressure $P_{f}$ of corroded pipelines should lie between that of intact pipelines $P_{f0}$ and that corresponding to the pipeline $P_{\min}$ at the location of the minimum residual wall thickness. $P_{f0}$ can be derived from the classic thin-walled cylinder formula: $P_{f0}=\sigma_{u}\times (2t/D)$, where $\sigma_{u}$ is the tensile strength. $P_{\min}$ can be approximated by substituting the residual wall thickness (t − d) into the above formula. Thus, a general form incorporating a defect influence function F can be constructed as: $P_{f}=P_{f0}\times F$ (geometric parameters).

The value range of the defect influence function F should be between 0 and 1. For an intact pipeline with no defects, F = 1; for a pipeline with extremely severe defects, F approaches (t − d)/t. Based on the aforementioned analysis, F should be primarily correlated with the relative depth and relative length $L/\sqrt{Dt}$, and should be able to reflect the nonlinearity of the depth effect and the saturation characteristic of the length effect. After trial calculation and comparison of various functional forms, the form shown in Equation (4) is finally determined.

(4) $$P_{f}=\frac{2\sigma_{b}t}{D}\left[1-\frac{d}{t}+\frac{d}{t}\left(a\cdot \exp\left(\frac{bL}{\sqrt{Dt}}\right)\right)\cdot {\left(1-\frac{d}{t}\right)}^{c}\right]$$

wherein a,b,c—undetermined coefficient.

### 4.2. Numerical Simulation Results

ABAQUS was employed to perform numerical simulations on 244 sets of corroded pipeline models with different parameter combinations, covering three steel grades and varying depths, lengths, widths and internal pressures, thus generating an extensive database of failure pressure. The undetermined coefficients in Equation (4) were fitted by the nonlinear least square method using the 1stOpt mathematical optimization software. In the fitting process, the finite element calculation results were taken as the target values, with the aim of minimizing the error between the model predicted values and the finite element results.

**Figure 15:**
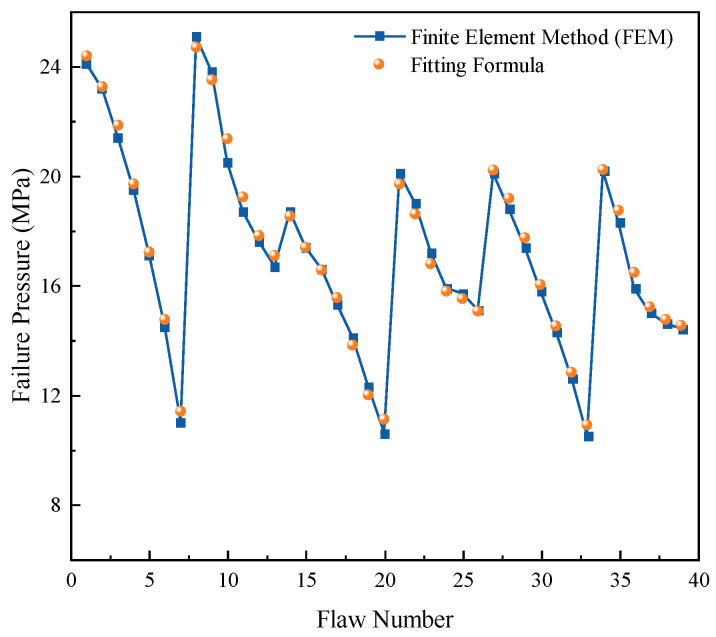
Comparison curve of finite element results and fitted calculations.

The fitting results are presented in Table 4. The coefficient of determination R2 reaches as high as 0.977, with the root mean square error (RMSE) being 0.911 MPa, which indicates an excellent fitting performance of the proposed model for the finite element data. Substituting the fitted coefficients into the formula yields the final failure pressure prediction model, as shown in Equation (5). Figure 15 depicts the comparison between the model-predicted values and the finite element calculated values of the 244 sets of data, where the data points are closely distributed on both sides of the 45° diagonal line, verifying the accuracy of the model within the range of the training data.

(5) $$P_{f}=\frac{2\sigma_{b}t}{D}[1-\frac{d}{t}+\frac{d}{t}(1.3411\cdot \exp(\frac{-0.3053L}{\sqrt{Dt}})\cdot {\left(1-\frac{d}{t}\right)}^{0.5212})]$$

### 4.3. Model Validation and Comparative Analysis

To verify the generalization ability and engineering practicability of the proposed model, 34 sets of full-scale burst test data from another independent literature were adopted for model validation. Meanwhile, these 34 sets of data were separately evaluated using the modified ASME B31G, DNV RP-F101, PCORRC and SY/T 6151 models, as well as a relatively new CUP model proposed by Shuai et al. [33].

**Figure 16:**
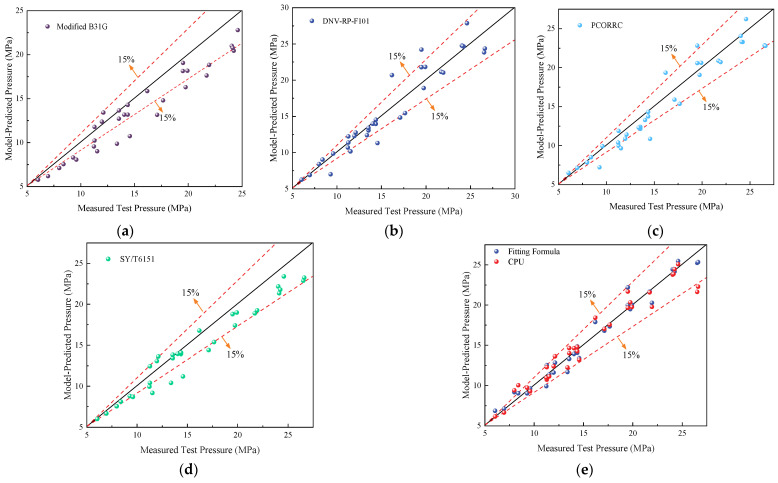
Comparison of failure pressure prediction models for corroded pipelines. (**a**) Modified ASME B31G; (**b**) DNV RP-F101; (**c**) PCORRC; (**d**) SY/T 6151; (**e**) fitted model and CUP model.

Figure 16 presents a comparison of the predicted values from each model with the experimental values. The two dashed lines in the figure represent the ±15% error range. It can be clearly observed that most of the predicted points of the ASME B31G and SY/T 6151 models fall within the conservative region below the diagonal line; the predicted points of the DNV RP-F101 and PCORRC models are relatively evenly distributed, yet some points still lie outside the 15% error band; in contrast, the predicted points of the CUP model and the proposed model are densely distributed around the 45° line, with nearly all points falling within the 15% error band.

Table 5 quantitatively compares the errors of each model from a numerical perspective. The mean absolute error of the proposed model is 6.81%, with a maximum error of 18.53% and a minimum error of 0.215%. Its mean error is slightly higher than that of the CUP model (6.46%) but significantly lower than those of the other four traditional methods. More importantly, the proposed model yields the smallest maximum error among all models, which indicates that its prediction results are more stable and less prone to severe deviations. The advantages of the proposed model lie in its simple form, which contains only three constants fitted from a large volume of data with clear physical meanings—corresponding to the intact pipeline strength, depth weakening factor and length weakening factor respectively. In addition, it is applicable to pipelines of different steel grades and requires no adjustment for different materials.

## 5. Conclusions

Through systematic finite element parametric analysis, this study conducts an in-depth investigation into the failure behavior of pipelines with single-point uniform corrosion defects under internal pressure loading, and establishes a high-precision prediction model for failure pressure. The main conclusions are as follows:

(1) Validation of the finite element model demonstrates that the ABAQUS model adopting the true stress–strain constitutive relation and plastic failure criterion can accurately predict the burst pressure of corroded pipelines, with a mean error of only 5.49% compared with full-scale test results, which lays a reliable foundation for the parametric study.
(2) The influences of geometric parameters of corrosion defects follow distinct regular patterns: corrosion depth is the dominant factor with a nonlinear effect, and the pipeline strength drops sharply when the relative depth d/t exceeds 0.6; the effect of corrosion length has a critical value (approximately $2\sqrt{Dt}$), beyond which the influence weakens; the effect of corrosion width on failure pressure is negligible within the range of 20° to 100°.
(3) Based on 244 sets of finite element data, a concise explicit prediction model for failure pressure was established (Equation (3)). This model enables failure pressure calculation using only the basic pipeline parameters ($D,t,\sigma_{u}$) and defect dimensions (d, L), and yields a mean error of 6.81% in comparison with 34 sets of independent test data, exhibiting higher accuracy than the commonly adopted industry standards.
(4) The research findings provide a directly applicable evaluation tool for engineering practice. It is recommended that in field inspections, the corrosion depth and length be measured with priority and high precision, while the corrosion width can be processed with simplification. For evaluation purposes, the proposed model can be first used to calculate the failure pressure, and then the maximum allowable operating pressure of the pipeline can be determined by combining with the safety factor, which provides a quantitative basis for maintenance decision-making.

This research can be further extended in the future to non-uniform corrosion, complex load combinations (e.g., internal pressure + bending moment), and pipelines with higher steel grades (e.g., X100, X120), so as to continuously improve the technical system for the integrity evaluation of corroded pipelines.

## Figures and Tables

**Table 1:** Geometric and mechanical properties of the models.

Pipe Grade	Outer Diameter (mm)	Wall Thickness (mm)	Yield Strength (MPa)	Tensile Strength (MPa)
X52	500	12	360	460
X65	762	17.5	435	530
X80	1219	19.8	550	620

**Table 2:** Full-scale burst test data of corroded pipelines.

Pipeline No.	Steel Grade	Pipe Diameter (mm)	Wall Thickness (mm)	Corrosion Depth (mm)	Corrosion Length (mm)	Corrosion Width (mm)	Yield Limit (MPa)	Tensile Limit (MPa)	Experimental Failure Pressure (MPa)
1	X65	762	17.5	4.4	200	50	467	576	24.11
2	X65	762	17.5	8.8	200	50	467	576	21.76
3	X65	762	17.5	13.1	200	50	467	576	17.15
4	X65	762	17.5	8.8	100	50	467	576	24.30
5	X65	762	17.5	8.8	300	50	467	576	19.80
6	X60	324	9.79	6.99	500	95.3	422.5	589.6	11.99
7	X60	324	9.74	7.14	528	95.3	422.5	589.6	11.30
8	X60	324	9.8	7.08	256	95.3	422.5	589.6	14.40
9	X60	324	9.66	6.76	306	95.3	422.5	589.6	14.07
10	X60	324	9.71	6.93	350	95.3	422.5	589.6	13.58
11	X80	459	8.00	3.75	40.0	32	589	731	24.2
12	X80	1219	19.89	15.41	605.72	—	550	625	9.30
13	X80	1219	19.89	7.44	605.72	—	550	625	17.7

**Table 3:** Calculation results of various industry standards.

Pipeline No.	Modified ASME B31G (MPa)	DNV-RP-F101 (MPa)	PCORRC (MPa)	SY/T 6151 (MPa)	Finite Element Method (MPa)
1	20.99	24.752	24.074	22.16	24.46
2	17.6019	21.11	20.874	18.871	21.44
3	13.117	14.786	15.858	14.366	16.25
4	20.421	24.621	23.287	21.775	24.11
5	16.274	18.876	19.036	17.393	18.48
6	12.33	12.241	10.832	13.033	11.03
7	11.73	11.315	9.924	12.397	10.75
8	13.13	13.91	13.699	13.883	13.49
9	13.14	13.922	13.238	13.892	13.19
10	12.67	13.001	12.11	13.389	12.14
11	20.758	24.687	23.305	21.33	23.32
12	8.239	6.92	7.155	8.743	8.75
13	14.766	15.395	15.328	15.34	17.14

**Table 4:** Fitting results of 1stOpt software.

Parameter	Value
a	1.3411
b	−0.3053
c	0.5212
Root Mean Square Error (RMSE)	0.9111
Correlation Coefficient R2	0.977
Sum of Squared Errors (SSE)	21.5827

**Table 5:** Error analysis of prediction models.

Error Analysis	Modified ASME B31G	DNV RP-F101	PCORRC	SY/T 6151	CUP Model	Proposed Prediction Model
Mean Error	11.946%	7.842%	8.333%	9.066%	6.459%	6.810%
Maximum Error	26.849%	23.836%	23.064%	22.686%	18.721%	18.528%
Minimum Error	0.200%	0.132%	0.476%	1.265%	0.497%	0.215%

## Data Availability

The original contributions presented in this study are included in the article. Further inquiries can be directed to the corresponding authors.
